# Transposable Elements Activity is Positively Related to Rate of Speciation in Mammals

**DOI:** 10.1007/s00239-018-9847-7

**Published:** 2018-05-31

**Authors:** Marco Ricci, Valentina Peona, Etienne Guichard, Cristian Taccioli, Alessio Boattini

**Affiliations:** 1 Department of Biological, Geological and Environmental Sciences, University of Bologna, Bologna, Italy; 2 Department of Ecology and Genetics, University of Uppsala, Uppsala, Sweden; 3 Department of Animal Medicine, Health and Production, University of Padova, Padova, Italy

**Keywords:** Speciation, Rate of speciation, Transposable elements, Cold genome, Relative rate of speciation, Mammals evolution

## Abstract

Transposable elements (TEs) play an essential role in shaping eukaryotic genomes and generating variability. Speciation and TE activity bursts could be strongly related in mammals, in which simple gradualistic models of differentiation do not account for the currently observed species variability. In order to test this hypothesis, we designed two parameters: the Density of insertion (DI) and the Relative rate of speciation (RRS). DI is the ratio between the number of TE insertions in a genome and its size, whereas the RRS is a conditional parameter designed to identify potential speciation bursts. Thus, by analyzing TE insertions in mammals, we defined the genomes as “hot” (high DI) and “cold” (low DI). Then, comparing TE activity among 29 taxonomical families of the whole Mammalia class, 16 intra-order pairs of mammalian species, and four superorders of Eutheria, we showed that taxa with high rates of speciation are associated with “hot” genomes, whereas taxa with low ones are associated with “cold” genomes. These results suggest a remarkable correlation between TE activity and speciation, also being consistent with patterns describing variable rates of differentiation and accounting for the different time frames of the speciation bursts.

**Electronic supplementary material:**

The online version of this article (10.1007/s00239-018-9847-7) contains supplementary material, which is available to authorized users.

## Introduction

Transposable elements (TEs) are DNA sequences that are able to move and replicate throughout the genome. They can be highly deleterious when inserted in genetic regions but they also have been a great source of genomic innovations (Richardson et al. 2015). For example, TEs play an important role in telomere maintenance (Farkash and Prak 2006), rewiring of transcriptional networks (Kunarso et al. 2010), regulation of gene expression (Chuong et al. 2016), ectopic recombination, and chromosomal rearrangements (Fedoroff 2012). Furthermore, TEs have been key contributors to evolution (Biemont 2010; Oliver et al. 2013; Kapusta et al. 2017) and led the insurgences of the V(D)J system of acquired immunity (Kapitonov and Jurka 2005; Koonin and Krupovic 2014) and mammalian placenta (Lynch et al. 2011). Given their huge impact on shaping genomes, TEs are also thought to influence differentiation (Huff et al. 2016) and speciation as proposed by the Epi-Transposon (Zeh et al. 2009), CArrier SubPopulation (Jurka et al. 2011), and TE-Thrust (Oliver and Greene 2012) hypotheses.

**Fig. 1:**
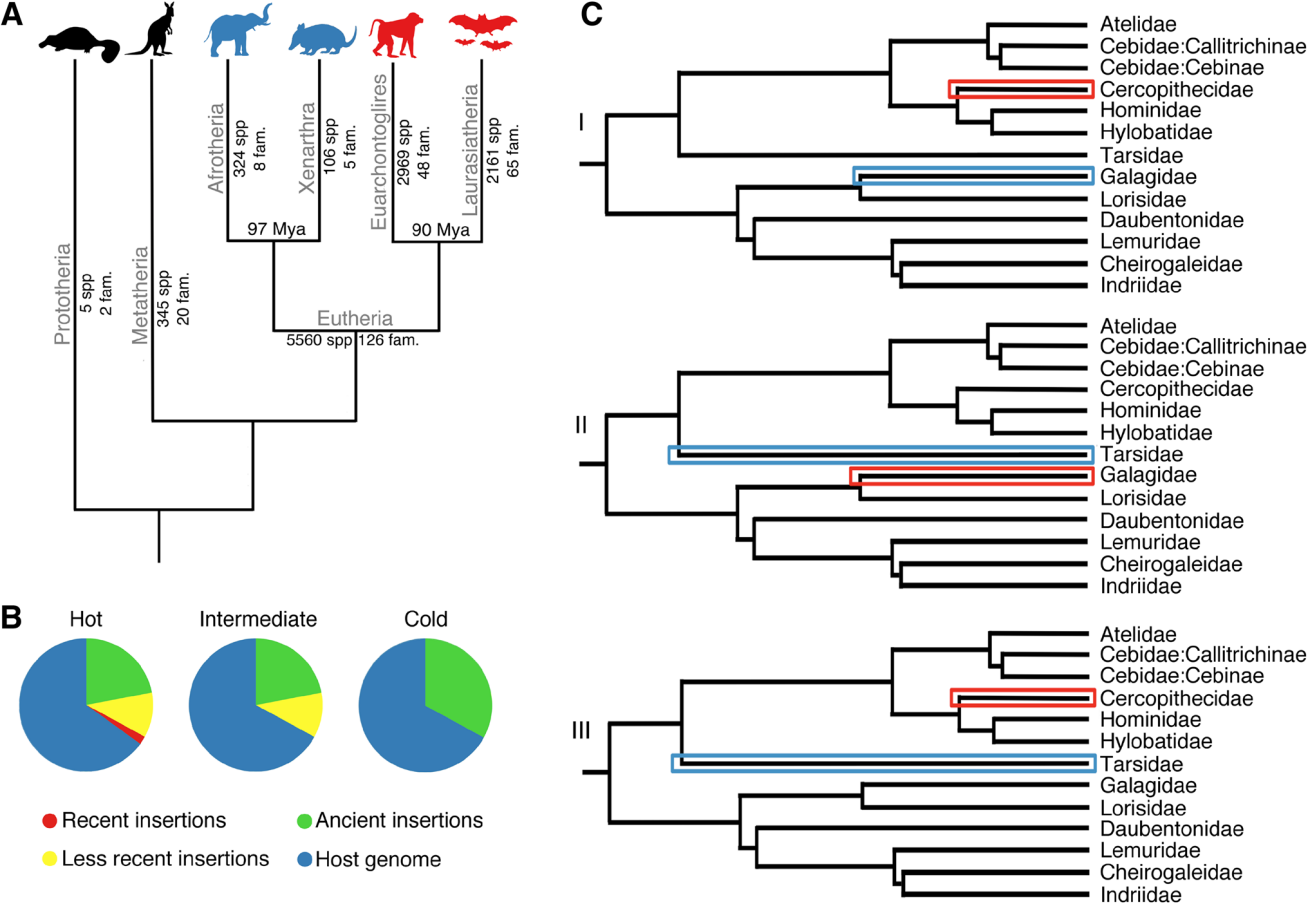
**a** Tree of mammals. Species abundance and phylogenetic relationships of the main mammalian clades. Putatively “hot” superorders of Eutheria (RRS(+)) are shown in red; putatively“cold” superorders (RRS(−)) are shown in blue. Animal icons made by Freepik from http://www.flaticon.com **b** Modelization of the Cold Genome hypothesis. “Hot” genomes contain a fraction of active, recently mobilized TEs (diverging less than 1% from their consensus sequence). “Intermediate” genomes contain a fraction of less recently mobilized TEs (diverging less than 5% from their consensus sequence). “Cold” genomes show ancient insertions with very low or absent activity. **c** Exemplified use of the relative rate of speciation (RRS) within the order Primates. I Galagidae, when compared to Cercopithecidae, are older and poorer in species, thus Galagidae: RRS(−), Cercopithecidae: RRS(+). II Galagidae, when compared to Tarsiidae, are younger and richer in species, thus Galagidae: RRS(+) and Tarsiidae: RRS(−). III Cercopithecidae when compared to Tarsiidae, are younger and richer in species, thus Cercopithecidae: RRS(+) and Tarsiidae: RRS(−). (Color code: RRS(+): red; RRS(−): blue). (Color figure online)

Phyletic gradualism (PG) (Charlesworth et al. 1982) and punctuated equilibria (PE) (Eldredge and Gould 1972) are the most important evolutionary theories for explaining speciation dynamics. According to PG, species continuously accumulate mutations that would eventually lead to differentiation and speciation (McPeek and Brown 2007). Instead, the PE theory suggests that rapid bursts of differentiation and speciation are alternated to “static” phases in which organisms do not significantly change (Eldredge and Gould 1972). Authors as Mattila and Bokma (2008) suggest that the PE theory should be taken into account for a more complete and accurate explanation of mammalian evolutionary dynamics. If TEs widely influenced speciation, as suggested by the previously reported hypotheses, it should be possible to find an association between the profiles of TE activity within genomes and patterns of speciation of organisms. For instance, the well-characterized mammalian phylogeny (Meredith et al. 2011) shows that the order Monotremata is the most ancient and the poorest in living species (Fig. 1a). Accordingly, the platypus genome, belonging to this taxon, should harbor the lowest number of recently mobilized TEs, which was actually demonstrated by Jurka et al. (2011). This specific case led to the hypothesis that Mammals with higher rates of speciation should show higher TE activity (“hot” genomes). Conversely, taxa with low rates of speciation should exhibit a lower TE activity (“cold” genomes; Fig. 1b, c).

In this study, we assess and test the association between speciation and TE activity in mammals, by taking into account both extant and extinct species. In fact, mammals have particularly detailed TEs annotation (Jurka et al. 2011) as well as a reliable phylogeny (Meredith et al. 2011) and abundant fossil records (Paleobiology Database 2018).

### Testing Phyletic Gradualism for Speciation and Extinction

In order to establish if the speciation rates varied or remained constant among the mammalian families, we have tested the prevalence of phyletic gradualism as a model of speciation. The main assumption of the PG theory is that genomes accumulate mutations and clades accumulate species constantly in time; therefore, older clades should be richer in species than younger ones. Older clades should also have accumulated more extinction events than younger ones. Correlation tests and linear regressions between clade age and species richness on all the 152 mammalian families (Table S1) were found to be statistically non-significant (*P* value 0.82) (Fig. S1). Correlation tests between the number of extinct species recorded for each clade (Table S2) and its age resulted in a non-significant association of those variables (*P* value 0.95) (Table S3). Furthermore, the extinction rate of a clade (calculated as extinct species/total species) does not show significant correlation with the clade’s age (*P* value 0.81) (Table S3). A linear regression model associating the rate of extinction and the clade age is, again, non-significant (*P* value 0.87) (Fig. S2, Table S3).

Thus, the PG model does not seem to describe mammalian evolution accurately, confirming previously reported results (Mattila and Bokma 2008).

### Rate of Speciation (RS), Life History Traits, and Density of Insertion (DI)

In order to evaluate TE activity in mammalian genomes, we took into account the data produced by the study of Jurka et al. (2011) (Table S4) that provide the number of insertions and the number of TE families (NF) diverging less than 1% and less than 5% (1NF, 5%NF) from their consensus sequences. The consensus sequence for a transposable element is the best approximation of the active element that gave rise to the different insertions in a genome. The divergence from consensus, on a large scale, is a proxy for the insertions’ age (Jurka et al. 2011). Therefore, we considered insertions diverging less than 1% as more recent, while those diverging less than 5% as older.

We designed a parameter called density of insertion (DI), which is the ratio between the number of TE insertions in a genome and its size, to summarize the level of TE activity in a genome. We calculated the DI at both divergence thresholds (1DI and 5%DI).

**Fig. 2:**
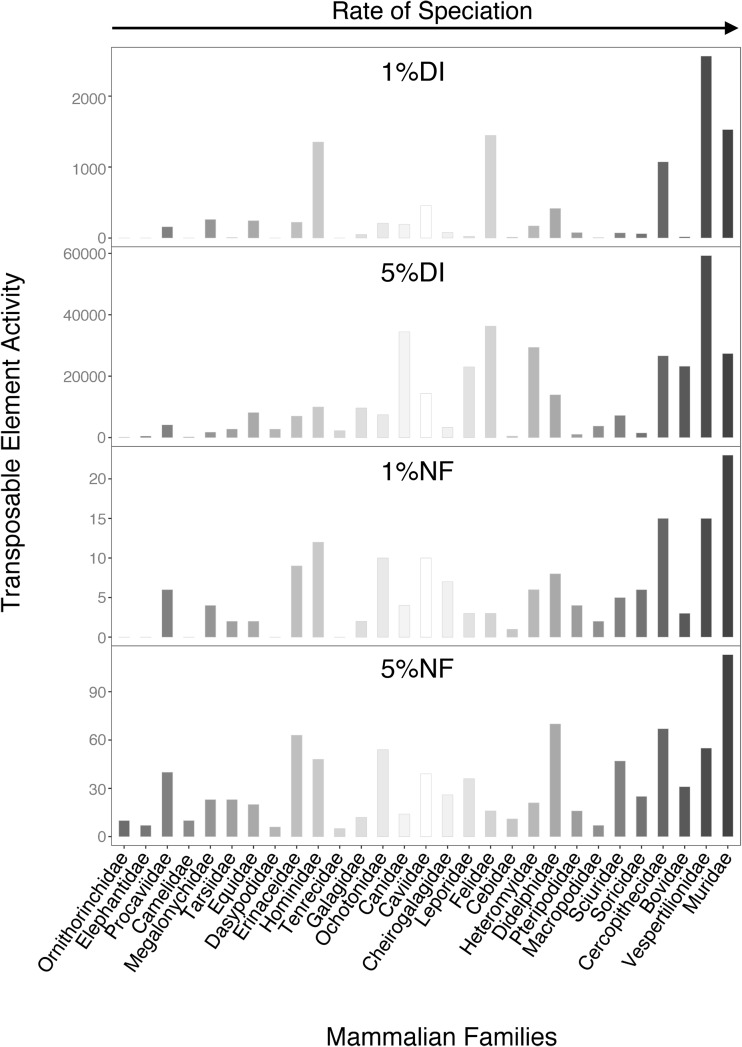
Relationship between the rate of speciation (RS) and TEs activity estimated according to the four considered parameters (1DI, 5DI, 1NF, 5%NF) in the 29 mammalian families of Eutheria. The families are arranged in the increasing order of RS (see also Table S11)

As for the rates of speciation (RS), we calculated them as the ratio between the number of extant species and the crown age (CA) of the taxon of interest (see “Materials and Methods” for details). We finally tested the hypothesis that TE activity is related with speciation patterns by estimating the association between NF, DI, and RS (Fig. 2) with Spearman correlation and linear regression models (Table S5A-S6A). Notably, all the parameters showed significant correlation with RS in the whole Mammalia class. In particular, linear models (Table S6A) showed positive regression coefficients and significant *P* values for all parameters except 5%DI. When extinct species are included in the RS calculation (Fig. S3), we obtained similar results. In fact, all the parameters show a significantly positive association with RS (TableS5B–S6B), with the exception of the linear regression model including 5%DI.

Since other factors may influence speciation processes, we tested the association between RS and two important life history traits, i.e., body mass and generation time (see Materials and Methods and Table S7).

The results of the Spearman correlation test suggest that high body mass is related to low RS (and vice versa, *P* value 0.021) and short generation time is related to high RS (and vice versa, *P *value 0.008) (Table S8).

Having ascertained that there is a potential relationship between life history traits and the rates of speciation, we tested if these non-genomic traits also show a correlation with TE activity. However, tests showed no correlation between these factors (Table S9–S10), with the only exception of generation time, shows a significant correlation with 5%DI. This association likely reflects the possible influence of meiosis frequency in the rate of TE accumulation in the long term. In fact, a higher number of generations imply that more TE insertion events are likely to have occurred and might have been transmitted from one generation to the other.

However, this effect seems to be only visible in a considerable amount of time and in a larger dataset of TE insertions (5%DI). We conclude that, while other parameters may impact TE dynamics, speciation rates in mammals are strongly and unambiguously related to TE activity, especially recent TE bursts (1%DI). Based on these results, it is tempting to speculate that the repertoire and activity of TEs in a given genome might contribute to its capability to diversify.

### Relative Rate of Speciation (RRS)

Under a non-gradualistic model of speciation and differentiation, RS, or the number of species alone, cannot identify and relatively locate finer adaptive radiation events within single taxonomical groups. In order to identify these events, we designed a new parameter called Relative rate of speciation (RRS) (Fig. 1c). RRS is a conditional parameter that compares a pair of taxa at the same hierarchical level (e.g., two families within the same order). Briefly, if one taxon of a given pair at the same time shows (1) a higher number of species and (2) a lower age compared to its paired taxon, then its RRS is positive (+) and putatively experienced a (relatively) recent speciation burst. Consequently, the other taxon has a negative RRS(−) and is experiencing a more static phase (Fig. 1c). If only one of the two conditions is met, there is no evidence of adaptive radiation/stasis for neither of the two taxa (RRS = 0) (see Materials and Methods and Supplementary Text 1). RRS can be applied at any taxonomical level on any monophyletic clade. In order to minimize the impact of external factors (such as differential generation time and genomic mutation rate of species belonging to distantly related taxa), we applied the RRS to mammalian families that belong to the same order and to mammalian superorders belonging to the same subclass. Given the genomic impact of transposable elements, we expect that genomes with higher TE activity (“hot”) should correspond to RRS(+) taxa, while RRS(−) taxa should have lower TE activity (“cold” genomes).

### RRS and DI Between Mammalian Families

**Fig. 3:**
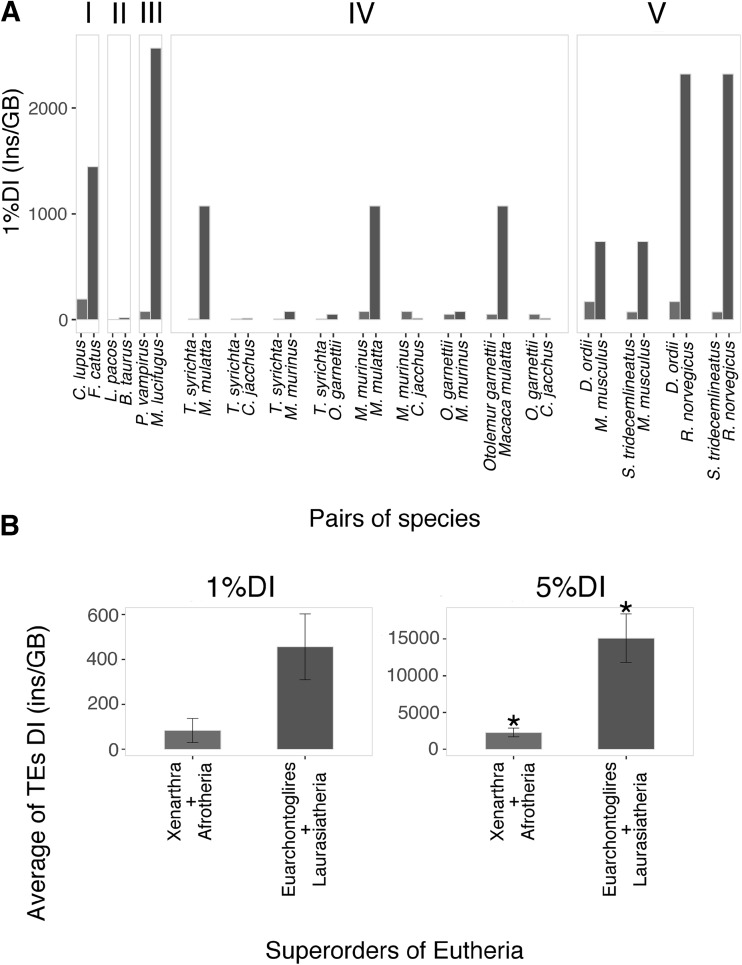
**a** 1%DI values in the 16 pairs of mammalian species which exhibit evidence of adaptive radiation/stasis. Blue bars: RRS(−) (putative “cold” genomes); red bars: RRS(+) (putative “hot” genomes). *I* Carnivora, *II* Cetartiodactyla, *III* Chiroptera, *IV* Primates, *V* Rodentia. **b** Comparison of the 1DI and 5%DI in the 4 superorders of Eutheria. Blue bars: RRS(−) (putative “cold” genomes); red bars: RRS(+) (putative “hot” genomes). ^*^P value < 0.05. (Color figure online)

At the lowest taxonomical level hereby considered (families within orders), we compared 15 mammalian species (encompassing six orders) arranged in 16 pairs (Table S11A). For each genome, we calculated the four parameters described above (1DI, 5%DI, 1NF, 5%NF; Table S10). We tested the association between putative “hot”/“cold” genomes (defined via RRS) and TE activity (DI and NF) with the paired Wilcoxon signed-rank test. All tests, excluding 5%DI, were significant (Table S12). The parameter with the highest confidence is 1%DI (Fig. 3a, Table S12). Using 1%DI, 14 out of 16 pairs matched the RRS results (Table S13, Supplementary Text 2). Furthermore, 11 pairs showed a difference in DI of at least one order of magnitude, up to almost 180-fold higher (*Macaca mulatta* vs. *Tarsius syrichta*).

RRS was estimated also considering the sum of the extant and extinct species for each taxon. By doing this, the number of possible comparisons increases from 16 to 20 (Table S11B). We tested the new list of paired species using the most descriptive among our four genomic parameters, i.e., 1%DI (Fig. S3). The Wilcoxon Signed-Rank test was highly significant (*P *value 6×10^−5^), with 18 out of 20 pairs following the expected trend. Thus, the inclusion of extinct species not only confirmed, but also enhanced the robustness of the association between TE activity and adaptive radiation events using RRS as a comparative strategy for speciation.

Overall, our RRS results suggest that, in mammals, the recent TE activity is associated with recent adaptive radiation. Therefore, we can conclude that the activity of TEs does not vary randomly within the mammalian phylogeny: on the contrary, the relative level of TE activity between two taxa is highly related to their relative ability to differentiate and speciate. In addition, 1%DI seems to be a more sensible parameter than NF for measuring recent TE activity (Supplementary Text 2).

### RRS and DI Between Placentalia Superorders

Next, we tested such association at a higher taxonomic level considering the Placentalia superorders of Afrotheria (A), Euarchontoglires (E), Laurasiatheria (L), and Xenarthra (X) (Fig. 3b, Table S14). According to RRS results, E and L showed RRS(+), thus putatively they are “hot” taxa, while A and X showed RRS(−), thus putatively they are “cold” taxa (Fig. 1a, Table S14). After averaging their respective DIs, we merged the putatively “hot” superorders (E and L, 22 species) and the putatively “cold” superorders (A and X, 5 species) and tested their association with DI as above (Supplementary Text 3). For both 1DI and 5%DI, “cold” superorders show an averaged DI more than threefold lower than “hot” superorders.

Differently from what is observed at the lower taxonomical level, 5%DI yields a significant difference between the two groups, while the 1%DI shows a non-significant association (Fig. 3b, Table S15). This discrepancy between the lower and higher taxonomical levels may be interpreted from an evolutionary point of view. In fact, 5%DI, which represents older accumulation of TE insertions, is the worst predictor of TE activity among the four considered parameters (1DI, 5%DI, 1NF, 5%NF) when studying recent speciation (Fig. 3a, Table S12). On the contrary, it is the best one when considering older macroevolutionary events, such as the differentiation of the four Eutheria superorders (Fig. 3b, Table S15). Hence, the divergence of the elements from their consensus does reflect, on average, their age (Jurka et al. 2011), and related adaptive radiation events.

## Discussion and Conclusions

With this study, we explored the relationship between TEs activity and speciation patterns in the mammalian lineage, highlighting their impact in the evolution of this phylum and in particular their possible association with bursts of speciation. Of course, we cannot exclude that taxonomical errors, such as the presence of cryptic species Bickford et al. (2007) or inappropriate descriptions of new taxa (Komarek and Beutel 2006; Zachos 2018), could propagate into our conclusions. However, compared to other clades, the mammalian taxonomy is undeniably quite well studied, and, additionally, the families here considered encompass a varied number of documented species (minimum: 7, maximum: 690, mean: 129 spp). We believe that these facts should minimize the probability of introducing biases in our analyses.

We started our study by showing that neither speciation nor extinction rates in mammals do follow a regular trend as predicted by phyletic gradualism, which therefore does not seem to be the best model to explain evolutionary patterns in the considered class.

Despite being influenced by a wide variety of biological processes and life history traits, speciation revealed a strong association with the activity of TEs. In particular, our analysis of TE content of the considered genomes showed that a high differentiation rate in a taxon is strongly related to an increased molecular activity of the TEs (Feiner 2016). Additionally, the measured TE parameters seem to be mostly unrelated to life history traits, such as body mass and generation time, suggesting that TE activity can autonomously affect differentiation processes. Therefore, TEs might play a major role in contributing to the genomic plasticity necessary for species differentiation.

Then, we designed a new parameter, the Relative Rate of Speciation (RRS), as a tool to identify finer adaptive radiation events that occurred within orders of the mammalian class. That way, we further strengthened the positive association between TEs and recent bursts of speciation. In fact, taxa that experienced a recent radiation (RRS(+)) were considered as “hot genomes” and showed a strong association with high TE activity, whereas taxa that are less likely to have experienced recent bursts of differentiation (RRS(−), “cold genomes”) generally show lower TE activity. In addition, we showed that TE insertions and their approximate occurrence times are consistent with clade differentiation estimates: older TE bursts are associated to older adaptive radiation events (origin of mammalian superorders), whereas novel TE bursts correlate to newer evolutionary phenomena (origin of mammalian families).

A number of recent studies suggests that TEs seem to be important for adaptive radiation (Carmi et al. 2011; Belyayev 2014; Elbarbary et al. 2016; Huff et al. 2016). TEs, which probably reach fixation during speciation events by genetic drift (Jurka et al. 2011), have been associated with a variety of relevant biological innovations (Richardson et al. 2015). TEs also have been shown to have a wide variety of functional/regulatory effects on the loci in which they insert (Goodier and Kazazian 2008; Cordaux and Batzer 2009), potentially generating new functional variants. Therefore, we could speculate that TE activity influences speciation patterns in mammals. However, since differential molecular evolution rates are positively correlated with punctuated patterns of speciation (Pagel et al. 2006), the observed TE insertion patterns may be interpreted as the outcome of the same processes that affect the rate of speciation at a population level. TE activity would then follow speciation events, and not the contrary, closely reflecting adaptive radiation (and promising to be ideal markers for phylogenetic analyses, as they are virtually homoplasy-free).

In conclusion, we hypothesize that TE activity is modulated in evolutionary time frames, producing alternations of insertional bursts and silencing (Muñoz-Lopez et al. 2011), which is consistent with the molecular processes that should occur as stated by the PE theory. Accordingly, recent studies show that young LINE-1 elements are mostly repressed via methylation while old TEs are regulated by the KRAB/KAP1 system (Castro-Diaz et al. 2015). Less-strongly silenced elements can produce bursts of insertions, potentially generating many new variants in a short time and leading to a “hot” state of the host genome, which is highly capable of responding to environmental stresses and selective pressures. While silencing mechanisms progressively inhibit TE activity (inducing a state of “cold” genome), their lack of contribution to molecular differentiation might lead to a relatively static phase that is consistent with variable-rate speciation patterns. Species harboring these static genomes could, in evolutionary time frames, be less likely to adapt to environmental changes, thus being more likely to become extinct. Both factors (i.e., TE activity/inactivity influencing speciation or extinction respectively) could account for species variability and phylogenetic relationships observed in present-day mammals.

Whether TE mobilization and accumulation of new insertions is the cause or the effect of adaptive radiation/speciation remains open for debate. However, the results presented in this study and the intrinsic characteristics of the mobilome activity suggest that TEs might have played an important role in the molecular differentiation of mammals and can continue to influence the evolution of their genomes.

## Materials and Methods

The numbers of species for all 152 mammalian families listed in the last mammalian phylogeny (Meredith et al. 2011) were retrieved from Catalogue of Life (http://www.catalogueoflife.com), whereas the number of extinct species from Paleobiology Database (https://paleobiodb.org). Their crown ages (CA) were estimated from the timed phylogenetic tree (Meredith et al. 2011). Data about body mass and generation time have been retrieved from Animal Diversity Web (https://animaldiversity.org). For each species, the generation time has been calculated as the sum of gestation time and the time from birth to the sexual maturity measured in months (namely, the time passed from a meiosis to the next). The body mass has been calculated as the median between the average body mass of males and females of each species measured in kg. Data about TE families and TE insertions in the genomes of the considered species were retrieved from Jurka et al. 2011. DI (Density of Insertion) is calculated according to the formula: DI = NI/GS, where NI is the total Number of Insertions (of elements at 1 or 5% divergence) and GS is the Genome Size in Gigabases. Rate of Speciation (RS) is calculated with the formula: RS = NS/CA, where NS is the number of extant species for the analyzed taxonomical family. RS calculation was then repeated taking extinct species into account (NS = number of extant species + number of extinct species for a given taxon).

The RRS (Relative Rate of Speciation) attribution is represented by the logical formulae:

$${\text{RRS}}1\left( + \right),{\text{ RRS}}2\left( - \right):\,{\text{NS}}1{\text{ }}>\,{\text{NS}}2\,\Lambda \,{\text{CA}}1{\text{ }}<{\text{ CA}}2,$$

$${\text{RRS}}1\left( - \right),{\text{ RRS}}2\left( + \right):{\text{ NS}}1{\text{ }}<{\text{ NS}}2\,\Lambda \,{\text{CA}}1{\text{ }}>{\text{ CA}}2.$$

If one of these conditions is false, there is no evidence of adaptive radiation events between the considered taxa therefore RRS = 0. RRS was applied on couples of families belonging to the same order (Table S11, Supplementary Text 2) and to the four superorders of Eutheria (Table S14, Supplementary Text 3). RRS determination was also repeated including extinct species in NS.

All Spearman correlation tests and linear regression models were performed in R (cor.test with method="spearman” and lm functions, respectively). We tested the correlation between putative “hot”/“cold” genomes and RRS(+/−) using the Wilcoxon Signed-Rank Test for both families and superorders (wilcox.test function). All statistical analyses and graphs were performed/produced with the R software.

## Electronic supplementary material

Below is the link to the electronic supplementary material.

Supplementary material Figures (PDF 2245 KB)

Supplementary material Tables (XLSX 40 KB)

Supplementary material Text (PDF 61 KB)
