# Supplementary material for: Transposable Elements Activity is Positively Related to Rate of Speciation in Mammals
Source: J Mol Evol. 2018 May 31;86(5):303–10. doi: 10.1007/s00239-018-9847-7 (PMC6028844; doi:10.1007/s00239-018-9847-7)
Supplement: Supplementary file 1 — Supplementary material Figures (PDF 2245 KB) [file 239_2018_9847_MOESM1_ESM.pdf]

Transposable elements activity is positively related to rate of speciation in mammals

Supplementary Figures

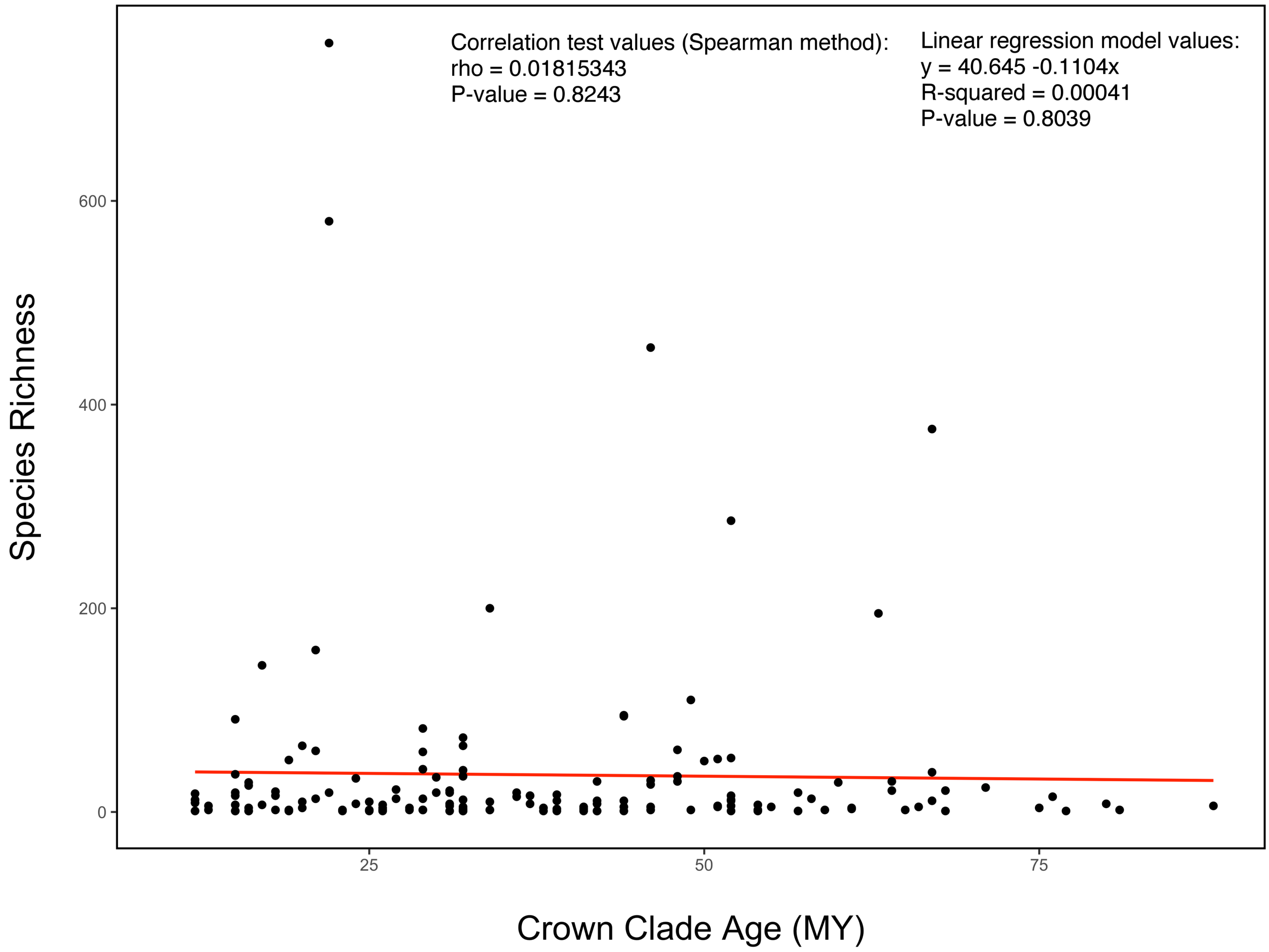

Figure S1. Relationship between the number of species and crown clade age of the 152 mammalian families. Each point represents a family of mammals. The regression line (red) and the statistics of the linear model and the corresponding correlation values are included. (See also **Table S1**).

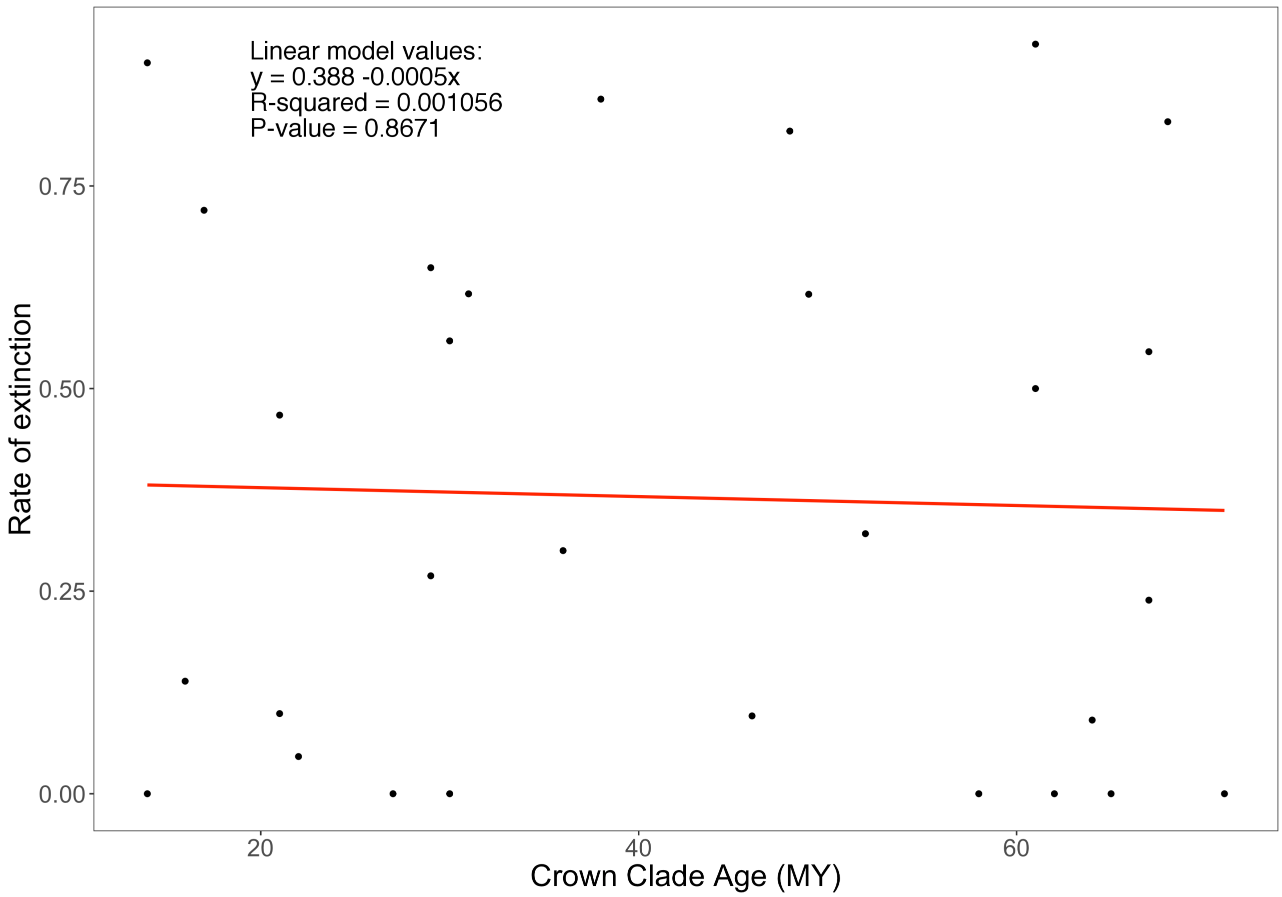

Figure S2. Relationship between crown clade age and the extinction ratio in 27 mammalian families. Each point represents a family of mammals. The regression line (red) and the statistics of the linear model.(See also **Table S2**).

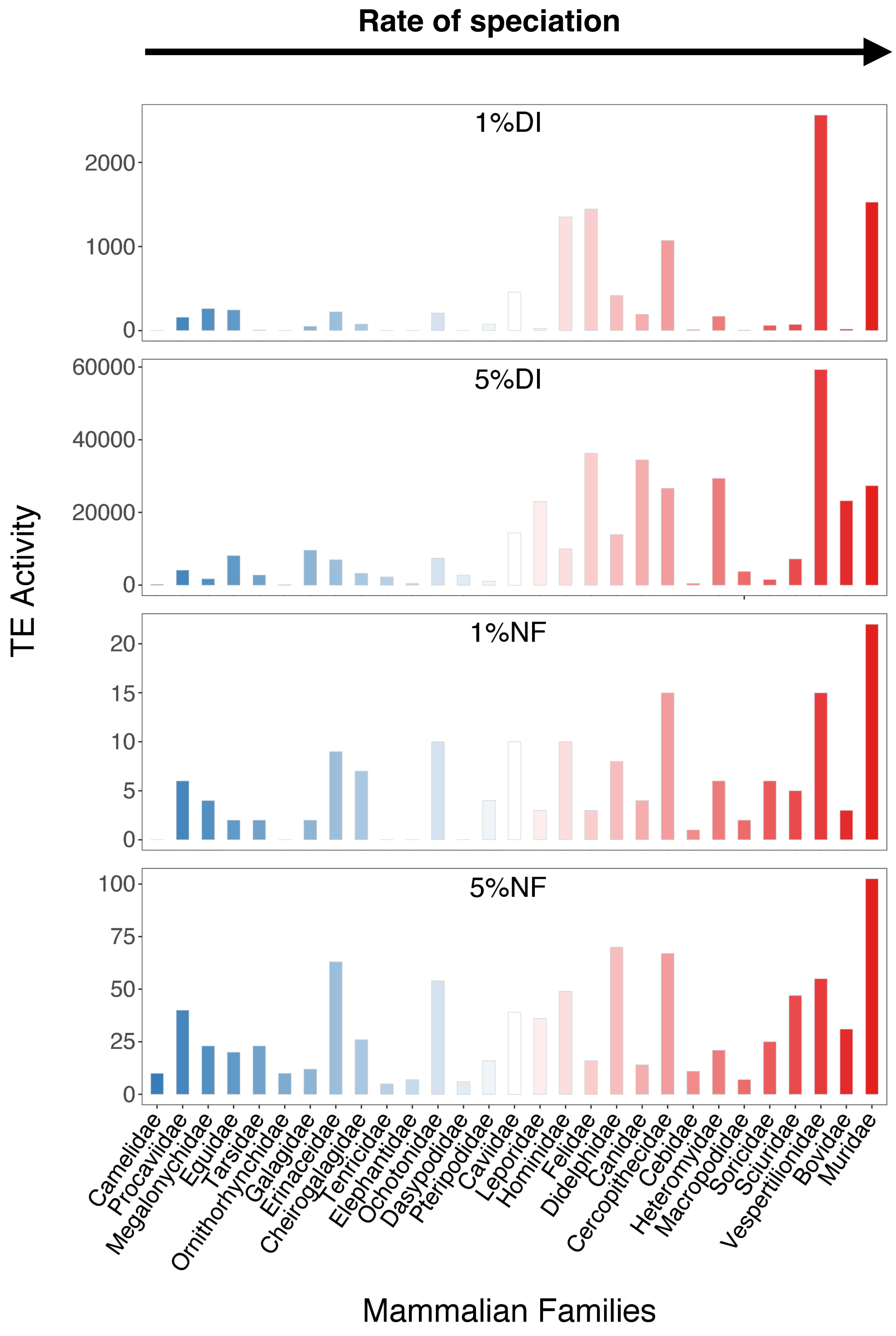

Figure S3. Relationship between the Rate of Speciation (RS) - calculated considering also the extinct species - and TEs activity estimated according to the four considered parameters (1%DI, 5%DI, 1%NF, 5%NF) in the 29 mammalian families of Eutheria. The families are arranged in increasing order of RS. (See also **Table S4**).

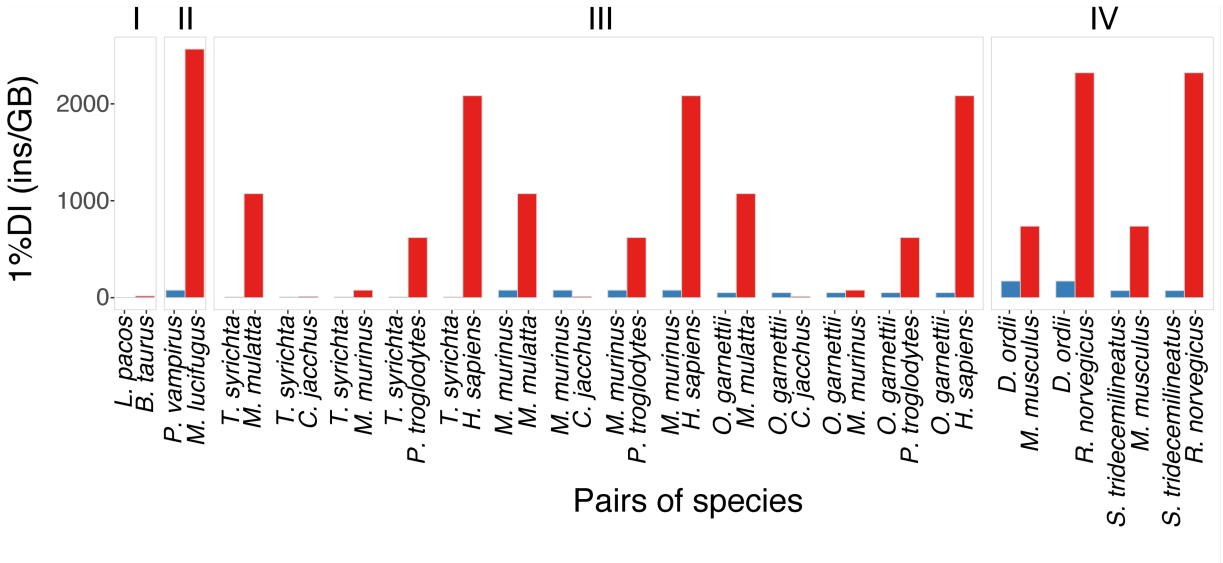

Figure S4. 1%DI values in 20 pairs of Mammal species which exhibit evidence of adaptive radiation/stasis. Blue bars: RRS (-) (putative "cold" genomes); red bars: RRS (+) (putative "hot" genomes); **I** Cetartiodactyla, **II** Chiroptera, **III** Primates and **IV** Rodentia orders.
