# Supplementary material for: Transposable Elements Activity is Positively Related to Rate of Speciation in Mammals
Source: J Mol Evol. 2018 May 31;86(5):303–10. doi: 10.1007/s00239-018-9847-7 (PMC6028844; doi:10.1007/s00239-018-9847-7)
Supplement: Supplementary file 3 — Supplementary material Text (PDF 61 KB) [file 239_2018_9847_MOESM3_ESM.pdf]

Transposable elements activity is positively related to rate of speciation in mammals

Supplementary Text

List of the abbreviations

DI: Density of Insertion (see Materials and Methods)

NF: Number of transposable element Families

PE: Punctuated Equilibria

PG: Phyletic Gradualism

RRS: Relative Rate of Speciation (see Materials and Methods)

TEs: Transposable elements

1) Use of the RRS: an extended example (Figure 1C)

In order to illustrate how the Relative Rate of Speciation (RRS) works, we provide a full example with three mammalian families.

The 36-million-year-old family Galagidae includes 19 extant species, whereas the 21-million-year-old family Cercopithecidae includes 159 species. Therefore, the Galagidae and Cercopithecidae families exhibits RRS (-) and RRS (+) respectively (see **Materials and Methods**). According to our hypothesis, Galagidae should include species with relatively "cold" genomes whereas the Cercopithecidae should include "hot" genomes (**Figure 1C section I**). Our prevision is confirmed by all DI results (see **Materials and Methods**). In particular, 1%DI is 51.3 ins/GB for Galagidae and 1073 ins/GB for Cercopithecidae (for full results see **Figure 3A section IV**, **Table S13**).

Next (**Figure 1C section II**) we proceeded to compare the families Galagidae (19 species, 36 My) and Tarsidae (11 species, 67 My). In this case Tarsidae has RRS (-), whereas the Galagidae has RRS (+) (putatively "colder" and "hotter" genomes, respectively). Also in this case the association between RRS (+/-) and "hot"/"cold" states agrees with 1%DI values (Tarsidae (-): 6,5 ins/GB; Galagidae (+): 51.3 ins/GB; see **Figure 3A section IV**, **Table S13**).Finally, we compared Cercopithecidae with Tarsidae (**Figure 1C section III**). This comparison yields RRS (+) for the Cercopithecidae and RRS (-) for Tarsidae. Once again the association with TE activity is significant when using 1%DI (**Figure 3A section IV**, **Table S12**).

Importantly, a taxon may show a positive RRS in one comparison and a negative one in another one, as shown above for the Galagidae case. These results can be explained considering the relative nature of RRS, which reflects the fact that an adaptive radiation event (i.e. a burst of speciation) can be identified only by means of comparisons with different taxa. In other words, as mentioned in the above example, the Galagidae family putatively experienced a speciation burst ("hot" genome, in our hypothesis) compared to Tarsidae. At the same time Galagidae are relatively less active ("cold" genome) when compared to Cercopithecidae, that instead experienced a bigger and more recent speciation burst. In fact, according to our hypothesis, the association between RRS (+/-) and "hot"/"cold" states (i.e. TE activity) can be interpreted also as a temporal series of adaptive radiation events.

When one of the two conditions defining a positive or negative RRS is not met, we conclude that there is no evidence of (relative) speciation bursts/stasis for the considered pair of taxa (RRS = 0). For example, let's consider the families Hominidae and Cercopithecidae. Hominidae shows a lower number of species (only 7 living species) when compared to Cercopithecidae (159 species); at the same time the family Cercopithecidae is 8 My older. Thus, Cercopithecidae could have accumulated a higher number of species as a consequence of their higher age (i.e. no evidence of speciation burst).

2) On the different outcomes of DI and NF parameters

Our Cold Genome hypothesis postulates that taxa with (relatively) high rates of speciation (RRS (+)) should show genomes with high TE activity ("hot" genomes), conversely, taxa with low rates of speciation (RRS (-)) should show genomes with low TE activity ("cold" genomes). In order to measure TE activity we proposed a new parameter called the Density of Insertion (DI) (see **Materials and Methods**). In this study we used both our new DI parameter as well as the Number of TE Families (NF) at 1% and 5% of divergence from their consensus sequences (1%NF and 5%NF, respectively), as proposed by Jurka et al. 2011.

Our results show that tests measuring the association between RRS(+/-) and "hot"/"cold" genomic states using the four TE activity parameters, yielded some significant differences (**Table S12**, **Table S13**). For example, with the 1%DI parameter, 14 out of 16 pairs follow the expected trend of association between DI values and RRS (**Table S13**). Among these, 11 pairs show a difference in DI of at least one order of magnitude, up to almost 180-fold higher in the pair *Macaca mulatta - Tarsius syrichta*. Despite two exceptions (*Microcebus murinus - Callithrix jacchus* and *Otolemur garnettii - Callithrix jacchus*). our analyses clearly suggest that 1%DI is strongly associated with adaptive radiations (see **Main Text**).

As for NF, in most cases this parameter is coherent with DI. However, in a few instances NF and DI yielded to opposite results.

The first case, is represented by the pair *Canis lupus* (RRS (-)) and *Felis catus* (RRS (+)). Compared to its paired species, *Canis lupus* features a higher number of specific TE families (1%NF = 4, 1%NF = 3, respectively), therefore NF does not agree with the "hot"/"cold" states. On the contrary, the density of mobile element insertions is lower in *Canis lupus* than in *Felis catus* (1%DI = 194 ins/GB and 1%DI = 1446 ins/GB, respectively), which is coherent with our hypothesis. The other case, in which 1%DI and 1%NF show discordance is the pair *Tarsius syrichta - Otolemur garnettii*. *Otolemur garnettii* (RRS (+)), in fact, shows an higher number of "recent" TEs (1%DI = 51 Ins/Gb) than *Tarsius syrichta* (RRS (-), 1%DI = 6 Ins/Gb) but the same number of TE families (1%NF = 2 for both species). A full discussion of the biological implications of the above exceptions goes beyond the scopes of the present work. As a consequence, 1%DI agreed with RRS (+/-) states, while 1%NF did not.

In conclusion, an higher number of active TE families does not necessarily reflect the relative "hotness" of the corresponding genome. Therefore, DI seems a more reliable measure of the impact of TE activity on a species genome. A genome can harbor a very diversified set of TE families, but this fact by itself does not imply their higher activity.

Table S13 lists the pairs of species that exhibit RRS (+/-) states, excluding RRS (0) ones. For each of the above pairs, we represented its coherence or discordance with the posits of the "Cold Genome" hypothesis using a green tick or a red cross respectively, for the four TE activity parameters. Interestingly, a) 1%DI shows the highest number of matches with RRS compared to all the other parameters (14/16, **Table S13**) ; b) discordances between 1%DI and RRS (i.e. the two above described cases) are replicated by all parameters. We conclude that 1%DI is the best predictor of genome "hotness"/"coldness" among the studied parameters.

3) On the choice of merging superorders data

In order to explore less recent macroevolutionary events, we tested our hypothesis with the split between the four superorders of Eutheria (Afrotheria, Euarchontoglires, Laurasiatheria and Xenarthra). According to RRS, Afrotheria and Xenarthra are putative "cold" clades (RRS (-)) while Euarchontoglires and Laurasiatheria as putative "hot" clades (RRS (+)). Then, we would obtain the following four pairs: Afrotheria - Euarchontoglires, Afrotheria - Laurasiatheria, Xenarthra - Euarchontoglires, Xenarthra - Laurasiatheria. However, we performed our analysis by merging Afrotheria and Xenarthra from one side ("cold" superorders) and Euarchontoglires with Laurasiatheria from the other side ("hot" superorders). This was done in order to overcome the low number of species present in the "cold" superorders (5 species, **Table S14**) compared to the "hot" superorders (22 species, **Table S14**). However, we believe that this merging of data should not affect the entity of the final result

Reference

Jurka J, Bao W, Kojima K. 2011. Families of transposable elements, population structure and the origin of species. Biology Direct 6:44.
